# Are Physical Activities Associated With Perceived Stress? The Evidence From the China Health and Nutrition Survey

**DOI:** 10.3389/fpubh.2021.697484

**Published:** 2021-08-03

**Authors:** Bing Cao, Yuxiao Zhao, Zhongyu Ren, Roger S. McIntyre, Kayla M. Teopiz, Xiao Gao, Ling Ding

**Affiliations:** ^1^Key Laboratory of Cognition and Personality, Faculty of Psychology, Ministry of Education, Southwest University, Chongqing, China; ^2^National Demonstration Center for Experimental Psychology Education, Southwest University, Chongqing, China; ^3^College of Physical Education, Southwest University, Chongqing, China; ^4^Mood Disorders Psychopharmacology Unit, University Health Network, Toronto, ON, Canada; ^5^Pharmacy Department, The Central Hospital of Jiangjin, Chongqing, China

**Keywords:** psychological stress, physical activity, preference, sports, sedentary, resiliency

## Abstract

Psychological stress is a negative affective state. The association between physical activity and psychological stress was commonly reported in previous researches. Few published studies with large sample sizes have explored such an association in Chinese population. The current research aims to assess the association between perceived stress and physical activity preferences, as well as the association between risk of high perceived stress and physical activity behaviors (e.g., sports-, transport-, occupational-related physical activity, sedentary activities and time in bed). The data were collected from the China Health and Nutrition Survey (CHNS) in 2015. Generalized linear models and logistic regression models were used to explore the association between perceived stress and physical activity. In total, 11,066 participants were included in the current analysis. For physical activity preferences, we found that “like” preference of all six mentioned domains of activities contributed to lower perceived stress (i.e., lower perceived stress scale −14 score, all β < 0 and *p* < 0.05). For physical activity behaviors, the results indicated that none/low intensity physical activity behaviors (e.g., sports-, occupational- related, sedentary) were associated with risk of high perceived stress, except that low intensity of transport-related activities seemed to be protective from high stress. However, the association between intensity of physical activity behaviors and perceived stress was not simply the higher the better. High intensity of all these physical activity behaviors was also associated with high perceived stress. Our findings suggest that positive preferences and moderate physical activity behaviors were associated with low perceived stress. The findings herein highlight the effect of regulating physical activity on perceived stress, as well as inform potential strategies to reduce psychological stress.

## Background

Psychological stress is a negative affective state, which is commonly associated with negative health outcomes, especially when it is chronic and unpredictable (1, 2). Accumulating evidence suggests that psychological stress is positively associated with some outcomes (e.g., smoking cigarettes, alcohol consumption) while negatively associated with other outcomes (e.g., physical activity) (3, 4). Several studies have reported that individuals may relieve or cope with stress *via* unhealthy yet rewarding behaviors, such as sedentary behavior and increased unhealthy eating behavior (5). In addition to changes in behaviors, chronic stress is also associated with disparate physiologic changes (e.g., increase in catecholamine release, cortisol dysregulation, and allosteric load) (6, 7). It is a highly replicated finding that chronic unpredictable stress predisposes and portends increased risk for obesity, metabolic syndrome, select forms of cancer, cardiovascular disease, mental disorders (e.g., major depressive disorder), as well as decreased quality of life and function (8, 9). The hazards posed by chronic, unpredictable stress provide the impetus for identifying potential risk factors for reduced health outcomes in stressed individuals.

Physical activity is defined as any movement of the body that results from skeletal muscles (10, 11). Epidemiological studies have reported on the bidirectional relationship between physical activity and stress, suggesting that daily physical activity may help to prevent, as well as improve, general physical and mental well-being in the context of stress (12, 13). Stults-Kolehmainen et al. reported that physical activity moderates the effect of excessive psychosocial stress and negative health outcomes. Additionally, they reported that the experience of stress attenuates efforts to be physically active (14). Extant literature also suggests that the interaction between physical activity and stress may be associated with several biological pathways, including but not limited to, aberrant brain connectivity, regulations in the hypothalamic-pituitary-adrenal axis, stimulation of a neurogenic processes, as well as alterations in the inflammatory system (15, 16). A recent publication pointed out that moderate level of physical activity is needed to retain a low level of stress during the COVID-19 pandemic lockdown (17). Physical activity interventions are also reported to tend to improve overall perceived stress mental health (18, 19). Numbers of previous basis found that higher physical activity levels are related to less perceived stress (20). Despite the overwhelming evidence suggesting an association between physical activity and mental health benefits (21, 22), whether there is a dose-response association between all the types of physical activities and mental health is still in debate. Some researchers argue that only leisure-time physical activity, but not transport-, occupational-, as well as sedentary activities are related to perceived stress and health (23). Moreover, the effect of physical activities on mental health seems not simply dose-response. Some researchers suggested that compared to highly vigorous physical activity, a moderate level would be more beneficial (24, 25). A recent study among preschool children reported that both low and high physical activity levels are related to unfavorable sleep characteristics. High physical activity is also found to be associated with increased post-traumatic stress disorder (PTSD) symptomatology among individuals aged 15 years and older in South Africa. Thus, the relationship between intensities of different types of physical activity and perceived psychological stress still needs further evaluation.

To the best of our knowledge, few published studies with large sample sizes have explored the association between physical activity and perceived psychological stress in the Chinese population. In addition, the exploration of the relationship between physical activity preferences and perceived stress have not been extensively reported (26). Against this background, the objective of the study herein is to examine the association between physical activity and perceived stress in a largescale sample of the Chinese population. We used the data from the China Health and Nutrition Survey (CHNS) to assess (1) the associations between perceived stress and physical activity preferences; (2) the associations between risk of high perceived stress and different categories of physical activity behaviors (e.g., sports-, transport-, occupational-related physical activity, sedentary activities, and time in bed). It was hypothesized that (1) perceived stress would be negatively associated with physical activity preferences; (2) both low and high intensity physical activity behaviors would be associated with the risk of high perceived stress.

## Methods

### Data Resource and Study Participants

We used the publicly available de-identified data from CHNS, which is an international collaborative project conducted by the National Institute for Nutrition and Health of the Chinese Center for Disease Control and Prevention and the University of North Carolina at Chapel Hill in the United States. The CHNS is a population-based longitudinal household survey which drew a sample using a multistage, random cluster sampling method. From 1989 to 2015, individual-level information on health, socioeconomic status, and social and family networks was collected in 11 waves (1989, 1991, 1993, 1997, 2000, 2004, 2006, 2009, 2011, 2015, and 2018, respectively). The first survey to incorporate Perceived Stress Scale (PSS)-Chinese version was conducted in 2015, and the most recent database in 2018 has not been released. As a result, the current study used data from the recently released 2015 wave of survey data from the CHNS. More detailed information about the CHNS project is available from the following URL: https://www.cpc.unc.edu/projects/china.

### Outcome Definition and Sample Selection Criteria

Perceived stress was measured with the Chinese version of the fourteen-item PSS (PSS-14). Accuracy of the Chinese version was ensured by translating the original PSS-14 from English to Chinese, and subsequently back into English (27). Each item was rated on a 5-point Likert-type scale, ranging from 0 = “never” to 4 = “very often.” The scale can be divided into two subscales: negative subscale (items 1, 2, 3, 8, 11, 12, and 14) and positive subscale (items 4, 5, 6, 7, 9, 10, and 13). The negative subscale asked frequency of negative incidents or feelings, such as “In the last month, how often have you felt that you were unable to control important things in your life?”. The positive subscale contained positively stated items, e.g., “In the last month, how often have you felt confident about your ability to handle your personal problems?”. Although the original PSS-14 contains a two-factor structure, this two-factor model did not fit well with the Chinese-validated version (27). Because of the weak correlation between these two factors (*r* = 0.2), scores of two subscales will be reported as a whole rather than separately to indicate the total perceived stress (28, 29), which ranges from 0 to 56. The one-factor structure showed satisfactory internal consistency (corresponding Cronbach's alpha = 0.83) (27). To obtain meaningful odds ratios (ORs), the variable was rescaled by the median of PSS-14 total score total as the unit of measure (i.e., low perceived stress and high perceived stress), which has also been used in previous studies [e.g., (30)].

In the CHNS questionnaire, the physical activity and inactivity preferences were assessed with the six activities listed as follows: Walking and Tai Chi, Sports (e.g., ping pong, badminton, tennis, soccer, basketball, volleyball), Body building, Watching TV, Computer/Video games, and Reading. The first three in the list are physical activity, and the other three are inactivity (31). For each activity and inactivity, the respondents were asked to rate their preference using the following responses: like very much, like somewhat, neutral, dislike somewhat, dislike very much, or does not participate. For physical activity participation, the respondents were asked to indicate whether they have participated in this activity selecting one of the options: “yes,” “no,” or, “unknown.” If respondents selected “yes” to a given item, they received a follow up question regarding participation time spent on that activity during a typical weekday or weekend day.

To create a measure of energy expenditure of physical activity behaviors, time spent in sport-related activities (i.e., martial arts; gymnastics, track and field; walking; soccer, basketball; badminton, volleyball), transport-related activities (i.e., by bus/subway; by bike; by foot; by car/taxi), and occupational-related activities (i.e., light activity; moderate activity; heavy activity) was multiplied by a specific metabolic equivalent of task (MET) intensity value. The final unit was MET-hours/week, which was the product of average number of hours spent per day participating in each physical activity and the MET score for that activity. The calculation method of MET was based on 2011 update of a major compendium of physical activities (32) and previous publication in Chinese population (33, 34). The MET value of each physical activity was listed in Supplementary Table 1. The sedentary activities (i.e., watching TV; VCR, VCDS, DVDS; video games; computer usage; reading, writing; surfing internet; joining chat rooms; playing computer games; watching videos/movies online; other sedentary activity) and time in bed were evaluated by the total time with the unit of hours/day.

We applied the sample selection from the dataset and merged the related files. Firstly, we merged the file of PSS, physical activity participation and preferences, and the basic demographic characteristics including: age, gender, education level, weight (kg), height (m), daily time in bed (hours), location, province, smoking status, alcohol consumption, and other necessary information. Then participants <18 years old who had indicated they do not know the level of their engagement in physical activities, or had missing data on stress outcomes, were excluded from analysis. Therefore, the final sample was comprised of 11, 066 (males = 5,870, females = 5,196).

### Statistical Analysis

Body mass index (BMI) was calculated by the weight (kg) divided by the square of height (m). MET rate time was calculated as follows: MET rate time = (time spent on each workday^*^5 + time spent on each weekend day^*^2)/7. Descriptive statistics was performed for the basic characteristics analysis. The continuous variables were summarized by means and standard deviations (SD), and the categorical variables were summarized by frequencies and proportions, respectively. For continuous variables, the independent samples *t*-test or one-way ANOVA was used for comparisons between two or more than two independent groups.

For categorical variables, statistical significance between various groups was tested using the χ^2^-test. The generalized linear models were used to explore the associations between perceived stress and physical activity preferences. Logistic regression models were used to explore the associations between risk of high perceived stress and physical activity behaviors. ORs and their 95% confidence intervals (CIs) were estimated using maximum likelihood methods. The variables of age, gender, community category (urban or rural) were included as covariates in the above models. The association was considered to be statistically significant if the 2-sided *p*-value is <0.05. All analyses were performed using Stata 15.0 (Stata Corp LP, College Station, TX, USA).

## Results

### Basic Characteristics of Subjects

Table 1 shows descriptive statistics for the variables of basic characteristics in included participants. The results were illustrated according to gender (i.e., male or female), age group (i.e., 18–40, 41–59, 60 years old and above), and community category (i.e., urban, rural). In total, 11,066 participants were included in the current study. The participants had a mean age of 52.0 (SD of 15.4) years and a mean BMI of 24.3 (SD of 4.2). Female participants made up 53.1% of the sample (*n* = 5,870). Most of the participants (60.7%) were from rural communities.

**Table 1:** Basic characteristics of included participants.

**Variables**	**Total**	**Gender**			**Age**				**Community category**		
		**Male**	**Female**	***p-value***	**18–40**	**41–59**	**≥60**	***p-value***	**Urban**	**Rural**	***p-value***
**Age** (years; Mean ± SD)	52.0 ± 15.1	52.2 ± 15.1	51.8 ± 15.1	0.156	31.4 ± 6.0	50.2 ± 5.4	68.3 ± 7.0	-	52.9 ± 15.3	51.4 ± 14.9	**<0.0001**
**Gender (** ***n*** **, %)**											
Male	5,196 (48.9)	-	-		1,176 (45.6)	2,237 (47.5)	1,783 (47.3)	0.625	2,007 (46.1)	3,189 (47.5)	0.155
Female	5,870 (51.1)	-	-		1,405 (54.4)	2,476 (52.5)	1,988 (52.7)		2,345 (53.9)	3,525 (52.5)	
**BMI** (kg/m^2^; Mean ± SD)	24.3 ± 4.2	24.4 ± 4.0	24.3 ± 4.3	0.134	23.5 ± 4.9	24.7 ± 3.8	24.3 ± 4.2	**<0.0001**	24.3 ± 4.0	24.3 ± 4.3	0.364
**Location (** ***n*** **, %)**											
Ruban	4,252 (39.3)	2,007 (46.1)	2,345 (53.9)	0.155	987 (38.2)	1,764 (37.4)	1,601 (42.5)	**<0.0001**	-	-	
Rural	6,714 (60.7)	3,189 (47.5)	3,525 (52.5)		1,594 (61.8)	2,949 (62.6)	2,170 (57.5)		-	-	
**Economic status (** ***n*** **, %)**											
East	5,487 (49.6)	2,595 (50.0)	2,892 (49.3)	0.778	1,257 (48.7)	2,268 (48.1)	1,962 (52.0)	**<0.0001**	2,375 (43.3)	3,112 (56.7)	**<0.0001**
Middle	3,754 (33.9)	1,750 (33.7)	2,004 (34.1)		868 (33.6)	1,748 (37.1)	1,138 (20.2)		1,232 (32.8)	2,522 (67.2)	
West	1,825 (16.5)	851 (16.4)	974 (16.6)		456 (17.7)	697 (14.8)	671 (17.8)		745 (40.8)	1,080 (59.2)	
**Education (** ***n*** **, %)**											
≤ Primary school	1,742 (18.6)	791 (16.2)	951 (20.4)	**<0.0001**	182 (7.2)	690 (16.3)	870 (33.4)	**<0.0001**	482 (12.3)	1,260 (23.2)	**<0.0001**
Secondary school	3,533 (37.7)	1,780 (37.9)	1,753 (37.6)		874 (34.7)	1,756 (41.4)	903 (34.7)		1,181 (30.1)	2,352 (43.3)	
≥High school	4,088 (43.7)	2,132 (45.3)	1,956 (42.0)		1,460 (58.0)	1,798 (42.4)	830 (31.9)		2,266 (57.7)	1,822 (33.5)	
**Time in bed per day** (hours; Mean ± SD)	7.8 ± 1.2	7.8 ± 1.2	7.8 ± 1.2	0.104	8.0 ± 1.0	7.8 ± 1.1	7.7 ± 1.2	**<0.0001**	7.6 ± 1.2	7.9 ± 1.2	**<0.0001**
**Smoking status (** ***n*** **, %)**											
Yes	2,493 (22.7)	2,724 (53.4)	113 (1.9)	**<0.0001**	543 (21.3)	1,183 (25.3)	767 (20.5)	**<0.0001**	867 (20.0)	1,626 (24.5)	**<0.0001**
No	8,472 (77.3)	2,380 (46.6)	5,748 (98.1)		2,010 (78.7)	3,485 (74.7)	2,976 (77.3)		3,459 (80.0)	5,013 (75.5)	
**Alcohol consumption (** ***n*** **, %)**											
Yes	2,906 (27.3)	2,612 (52.1)	294 (5.2)	**<0.0001**	651 (26.6)	1,409 (31.0)	846 (23.3)	**<0.0001**	1,125 (26.5)	1,781 (27.9)	0.117
No	7,726 (72.7)	2,405 (47.9)	5,321 (94.8)		1,795 (73.4)	3,137 (69.0)	2,906 (27.3)		3,120 (73.5)	4,606 (72.1)	
**PSS-14 score** (Mean ± SD)	22.9 ± 6.1	22.8 ± 6.1	23.1 ± 6.2	**0.023**	23.0 ± 5.8	22.9 ± 6.1	23.0 ± 6.3	0.837	22.3 ± 6.1	23.4 ± 6.1	**<0.0001**

*PSS, Perceived Stress Scale; BMI, Body mass index*.

*Significant p-values (< 0.05) are in bold*.

The average time in bed per day of the participants was 7.8 h (SD of 1.2). The differences of education levels were statistically significant in different gender, age group and community category (all *p* < 0.0001). Reports of smoking and alcohol consumption were significantly different between males and females, as well as different age groups (both *p* < 0.0001). No significant difference of reported smoking behavior was found between participants from urban and rural communities (*p* = 0.117). Female (*p* = 0.023) and participants from rural communities (*p* < 0.0001) reported higher perceived stress.

### Association Between Perceived Stress and Physical Activity Preferences

We conducted an analysis to explore the associations between perceived stress and physical activity and inactivity preferences. The means and SDs were also calculated in each group of each activity preference. The “Neutral” activity preference option was considered as a reference value. It was observed from the models that the PSS-14 score was negatively correlated with “like somewhat,” “like very much” in all six investigated domains of physical activity and inactivity preferences both before and after adjusting the potential confounders of age, gender and community category (all β < 0 and *p* < 0.05). Positive correlations were also found between PSS-14 scores with “does not participate” in walking/Tai Chi, watching TV, computer/video games and reading (all β > 0 and *p* < 0.05). Additionally, the results indicated that PSS-14 score was negatively correlated with “dislike” of walking/Tai Chi, computer/video games and reading. The details are shown in Table 2.

**Table 2:** Association between perceived stress and physical activity preferences.

**Variables**	***n***	**Mean ± SD**	**Crude**			Adjusted^*^		
			**β (95%CI)**	***z***	***p-value***	**β (95%CI)**	***z***	***p-value***
**Walking, Tai Chi**								
Dislike very much	526	23.6 ± 6.3	0.228 (−0.890, 0.228)	−1.16	0.245	−0.448 (−1.007, 0.110)	−1.57	0.116
Dislike somewhat	3,188	23.2 ± 6.2	−0.403 (−1.005, −0.403)	−4.59	**<0.0001**	−0.772 (−1.073, −0.471)	−5.03	**<0.0001**
Neutral	3,004	23.9 ± 5.5	REF			REF		
Like somewhat	3,863	22.0 ± 6.2	−1.667 (−2.242, −1.667)	−13.32	**<0.0001**	−1.887 (−2.178, −1.596)	−12.72	**<0.0001**
Like very much	249	19.4 ± 6.9	−3.741 (−5.301, −3.741)	−11.36	**<0.0001**	−4.37 (−5.155, −3.586)	−10.93	**<0.0001**
Does not participate	235	25.5 ± 5.9	2.337 (0.736, 2.337)	3.76	**<0.0001**	1.357 (0.556, 2.158)	3.32	**0.001**
**Sports (ping pong, badminton, tennis, soccer, basketball, volleyball)**								
Dislike very much	511	23.5 ± 6.6	0.862 (−0.263, 0.862)	1.04	0.296	0.159 (−0.403, 0.721)	0.55	0.580
Dislike somewhat	4,643	23.1 ± 6.2	0.209 (−0.311, 0.209)	−0.39	0.699	−0.176 (−0.437, 0.085)	−1.32	0.186
Neutral	3,879	23.2 ± 5.8	REF			REF		
Like somewhat	1,636	21.8 ± 6.2	−0.959 (−1.663, −0.959)	−7.3	**<0.0001**	−1.187 (−1.541, −0.833)	−6.57	**<0.0001**
Like very much	79	20.1 ± 6.8	−1.737 (−4.453, −1.737)	−4.47	**<0.0001**	−2.831 (−4.186, −1.475)	−4.09	**<0.0001**
Does not participate	317	23.6 ± 6.6	1.189 (−0.206, 1.189)	1.38	0.167	0.472 (−0.225, 1.170)	1.33	0.185
**Body Building**								
Dislike very much	451	23.6 ± 6.6	1.001 (−0.187, 1.001)	1.34	0.179	0.277 (−0.317, 0.871)	0.91	0.361
Dislike somewhat	4,616	23.0 ± 6.3	0.073 (−0.444, 0.073)	−1.41	0.160	−0.296 (−0.556, −0.037)	−2.24	0.025
Neutral	3,972	23.2 ± 5.7	REF			REF		
Like somewhat	1,614	22.0 ± 6.2	−0.830 (−1.536, −0.830)	−6.57	**<0.0001**	−1.075 (−1.428, −0.723)	−5.97	**<0.0001**
Like very much	69	20.2 ± 7.4	−1.485 (−4.388, −1.485)	−3.96	**<0.0001**	−2.700 (−4.148, −1.252)	−3.66	**<0.0001**
Does not participate	343	23.8 ± 6.5	1.245 (−0.101, 1.245)	1.67	0.096	0.516 (−0.157, 1.188)	1.50	0.133
**Watching TV**								
Dislike very much	135	24.1 ± 6.0	1.411 (−0.687, 1.411)	0.68	0.499	0.265 (−0.781, 1.310)	0.50	0.620
Dislike somewhat	821	23.7 ± 6.4	0.427 (−0.511, 0.427)	−0.18	0.861	−0.119 (−0.587, 0.350)	−0.50	0.620
Neutral	3,019	23.7 ± 5.6	REF			REF		
Like somewhat	6,610	22.5 ± 6.3	−0.905 (−1.429, −0.905)	−8.73	**<0.0001**	−1.176 (−1.438, −0.915)	−8.83	**<0.0001**
Like very much	375	21.6 ± 6.4	−1.485 (−2.79, −1.485)	−6.42	**<0.0001**	−2.045 (−2.697, −1.394)	−6.16	**<0.0001**
Does not participate	105	25.8 ± 3.9	3.258 (0.891, 3.258)	3.44	**0.001**	1.915 (0.735, 3.095)	3.18	**0.001**
**Computer/video games**								
Dislike very much	835	22.9 ± 6.6	−0.347 (−1.296, −0.347)	−3.39	**0.001**	−0.769 (−1.250, −0.288)	−3.14	**0.002**
Dislike somewhat	5,074	22.7 ± 6.3	−0.774 (−1.351, −0.774)	−7.22	**<0.0001**	−1.13 (−1.425, −0.836)	−7.52	**<0.0001**
Neutral	2,575	23.8 ± 5.5	REF			REF		
Like somewhat	1,970	22.3 ± 6.0	−1.11 (−1.824, −1.11)	−8.06	**<0.0001**	−1.477 (−1.843, −1.112)	−7.92	**<0.0001**
Like very much	185	21.3 ± 6.3	−1.579 (−3.393, −1.579)	−5.37	**<0.0001**	−2.37 (−3.279, −1.461)	−5.11	**<0.0001**
Does not participate	426	25.0 ± 5.7	1.910 (0.663, 1.910)	4.05	**<0.0001**	1.184 (0.556, 1.813)	3.69	**<0.0001**
**Reading**								
Dislike very much	526	23.5 ± 6.5	0.679 (−0.434, 0.679)	0.43	0.667	−0.001 (−0.560, 0.558)	0	0.996
Dislike somewhat	4,676	23.0 ± 6.3	−0.166 (−0.699, −0.166)	−3.18	**0.001**	−0.580 (−0.851, −0.309)	−4.2	**0.001**
Neutral	3,448	23.4 ± 5.5	REF			REF		
Like somewhat	1,922	21.7 ± 6.4	−1.419 (−2.096, −1.419)	−10.18	**<0.0001**	−1.581 (−1.922, −1.240)	−9.08	**<0.0001**
Like very much	158	20.0 ± 6.7	−2.420 (−4.355, −2.420)	−6.86	**<0.0001**	−3.134 (−4.101, −2.166)	−6.35	**<0.0001**
Does not participate	335	25.2 ± 5.5	2.461 (1.100, 2.461)	5.13	**<0.0001**	1.606 (0.924, 2.289)	4.61	**<0.0001**

* *Adjusted β and 95%CI were calculated by adjusting for the potential confounders including age, gender, community category*.

*REF, reference; MET, metabolic equivalent of task*.

*Significant p-values (< 0.05) are in bold*.

### The Association Between Risk of High Perceived Stress and Physical Activity Behavior

We rescaled the variable of PSS-14 total scores into low and high perceived stress by the median of their values. Table 3 shows the logistic regressions assessing the associations between risk of high perceived stress and physical activity behaviors. For sports-related physical activity, both none and high (>28) MET-hours/week were associated with risk of high perceived stress (AOR = 1.381, 95%CI: 1.124, 1.696; *p* = 0.002; AOR = 1.255, 95%CI: 1.095, 1.438; *p* = 0.001, respectively).

**Table 3:** The association between risk of high perceived stress and physical activity.

**Variables**	***n***	**UOR (95%CI)**	***z***	***p-value***	AOR^*^ (95%CI)	***z***	***p-value***
**Sports-related physical activity (MET-hours/week)**							
None	513	1.286 (1.050, 1.574)	2.43	**0.015**	1.381 (1.124, 1.696)	3.07	**0.002**
Low (≤ 14)	758	1.022 (0.855, 1.222)	0.24	0.810	1.009 (0.843, 1.208)	0.10	0.919
Moderate (>14 to 28)	1,711	REF			REF		
High (>28)	1,951	1.193 (1.042, 1.365)	2.56	**0.010**	1.255 (1.095, 1.438)	3.26	**0.001**
**Transport-related physical activity (MET-hours/week)**							
Low (≤ 7)	1,594	0.874 (0.755, 1.012)	−1.80	0.072	0.858 (0.741, 0.995)	−2.03	**0.042**
Moderate (>7 to 21)	1,414	REF			REF		
High (>21)	932	0.834 (0.704, 0.988)	−2.09	**0.036**	0.859 (0.724,1.020)	−1.74	0.082
**Occupational-related physical activity (MET-hours/week)**							
Low (≤ 60)	1,979	1.372 (1.217, 1.547)	5.16	**<0.0001**	1.366 (1.210, 1.541)	5.06	**<0.0001**
Moderate (>60 to 180)	2,573	REF			REF		
High (>180)	544	1.214 (1.004, 1.467)	2.00	**0.045**	1.134 (0.935, 1.376)	1.28	0.200
**Sedentary activity per day (hours)**							
≤ 2	4,116	1.571 (1.391, 1.774)	7.27	**<0.0001**	1.489 (1.315, 1.685)	6.3	**<0.0001**
>2 to 4	3,248	1.194 (1.052, 1.355)	2.75	**0.006**	1.162 (1.023, 1.320)	2.31	**<0.0001**
>4 to 6	1,521	REF			REF		
>6 to 8	570	1.035 (0.847, 1.265)	0.34	0.736	1.038 (0.849, 1.270)	0.36	0.717
>8	795	1.525 (1.281, 1.817)	4.73	**<0.0001**	1.453 (1.218, 1.732)	4.16	**<0.0001**
**Time in bed per day (hours)**							
<6	326	1.07 (0.857, 1.336)	0.59	0.552	1.404 (0.862, 1.351)	0.67	0.504
≥6 to 10	1,0485	REF			REF		
>10	247	1.341 (1.042,1.726)	2.28	**0.023**	1.080 (1.089,1.810)	2.62	**0.009**

* *AOR and 95%CI were calculated by adjusting for the potential confounders including age, gender, community category*.

*UOR, univariate odds ratio; AOR, adjusted odds ratio; REF, reference; MET, metabolic equivalent of task*.

*Significant p-values (< 0.05) are in bold*.

After adjusting age, gender and community category, low transport-related physical activity was associated with low perceived stress (AOR = 0.858, 95%CI: 0.741, 0.995; *p* = 0.042). Low occupation-related physical activity was associated with risk of high perceived stress. Significant associations between both low and high sedentary activity per day and risk of high perceived stress were also reported. Additionally, time in bed >10 h/day was associated with risk of high perceived stress (AOR = 1.404, 95%CI: 1.089, 1.81; *p* = 0.009).

## Discussion

The overarching goal of the study herein was to characterize the association between perceived stress and physical activity preferences and behaviors. By using publicly available data from CHNS in this analysis, we evaluated the association between perceived stress and six physical activity and inactivity preferences, as well as the association between risk of high perceived stress and physical activity behaviors in 11,066 participants from the Chinese population. For the physical activity preferences, we found that “like” preference of all six mentioned domains of activities is associated with lower perceived stress (i.e., higher PSS-14 score). Moreover, the preferences of “dislike” and/or “does not participate” in some activities (e.g., walking/Tai Chi, computer/video games and reading) were associated with higher perceived stress.

For the physical activity behaviors, the results indicated that both no physical activity and low intensity of physical activities (such as sports-, occupational- related, sedentary, time in bed) were associated with risk of high perceived stress. However, low intensity of transport—related activities were protective from high stress. Our results herein suggest that greater intensity of physical activities may be beneficial in the prevention of high psychological stress.

To our knowledge, relatively few prior studies have explored the relationship between physical activity as well as inactivity preferences and psychological stress in a largescale sample of the Chinese population. In general, the individuals who “like” all six domains of activities in the preference questionnaire have lower perceived stress than those who “does not participate in” or “dislike.” Moreover, reports of analyses conducted on CHNS data indicated that leisure time physical activity preference was a significant predictor of actual leisure time physical activity behavior (26).

A separate study of patients with lung cancer revealed that physical activity preferences were associated with social support and self-efficacy (35). According to our existing evidence, positive activity preferences are associated with low perceived stress, especially in activities related to sports (i.e., walking, Tai Chi, sports, body building). However, potential underlying psychological mechanisms require further investigation.

Previous studies have assessed the effect of physical activity participation on psychological stress, and have reported that high-intensity physical activity is better for stress prevention and management than low-intensity leisure time physical activity (20, 36). One possible explanation is that prior physical activity before a stressful situation can reduce cortisol concentrations during psychosocial stress and furtherly reduce the impact of stress on health (37). However, from our current analysis, we found that excessive high-intensity sports-related physical activities were not correlated with lower perceived stress. In fact, too much exercise may backfire and lead to higher level of stress. Since it can be observed in our study that both none participation as well as high MET-hours/week sports-related physical activity increased the risk of high perceived stress when compared with moderate intensity of physical activities, our findings supported the U-shaped relationship between exercise intensity and psychological stress.

We also observed that low intensity of transport-related physical activity had a protective effect on perceived stress. Additionally, our results suggest that low intensity MET-hours/week of occupational-related activities were related to increased risk of high perceived stress when compared with the moderate intensity. This is in accordance with additional reports of physical activity as a protective factor for psychological stress (38).

It has been reported that psychosocial stress is one risk factor for sedentary behavior in pregnant women (39); however, additional studies have reported that not all forms of sedentary activities are linked to perceived stress (40). Moreover, it has been reported that sleep deprivation may be a risk factor for psychological stress (41, 42), although we did not find the association between lack of sleep (i.e., <6 h/day) and high perceived stress in our sample. From our findings, we also determined that spending too much time in bed (i.e., >10 h/day) may be a risk factor for perceived stress. Although there are few studies focusing on the relationship between excessive sleep or hypersomnia and psychological stress, previous studies have found that a longer time spent in bed is associated with negative mental health outcomes, including but not limited to, depressed mood and suicidality (27, 43, 44). It was separately reported in a group of civil servants in Japan that that a U-shaped association exists between time in bed with poor sleep and psychosocial stress (45).

Overall, our study revealed that the relationship between perceived stress and physical activity preferences as well as behaviors were not linear associations. There should be a suitable reference range for each activity to reduce the perceived stress. Our findings herein provide preliminary evidence for formulating a reasonable reference range to regulate physical activity. Further longitudinal researches are needed to clarify the influence of specific physical activity on alteration of perceived stress.

### Limitations

Several methodological aspects of our study may affect inferences and interpretations of our findings. Firstly, as this analysis was cross-sectional, the results can only be concluded as associations with no implication of causality or directionality of the effect. Secondly, the CHNS delimited its assessment of perceived stress levels one time with PSS-14, which is a self-reported scale. Thirdly, because some important variables (e.g., the frequency of physical activity behaviors) were not available and some data of individuals were missing in our download version, our current analysis was limited to the accessible data. Notwithstanding the limitations, the results of our study provide an important basis for future intervention to reduce perceived stress and improve quality of life and well-being in a large-scale population.

## Conclusion

In summary, our results indicated that positive attitudinal preferences to select activities as well as moderate physical activity were associated with low perceived stress in a large-scale sample of the Chinese population. The findings herein also provide an empirical basis for prescribing exercise intensity at a population level and provide impetus to explore protective biological factors mediating the health outcomes observed.

## Data Availability Statement

The datasets presented in this study can be found in online repositories. The names of the repository/repositories and accession number(s) can be found below: https://www.cpc.unc.edu/projects/china.

## Ethics Statement

The studies involving human participants were reviewed and approved by University of North Carolina at Chapel Hill. The patients/participants provided their written informed consent to participate in this study.

## Author Contributions

LD and BC conceived and designed the study. BC and ZR performed the statistical analysis. BC, LD, and XG contributed to the discussion. YZ, RM, KT, LD, and ZR revised the paper. All authors have read and approved the final version of this article.

## Conflict of Interest

RM has received research grant support from CIHR/GACD/Chinese National Natural Research Foundation; speaker/consultation fees from Lundbeck, Janssen, Purdue, Pfizer, Otsuka, Takeda, Neurocrine, Sunovion, Bausch Health, Novo Nordisk, Kris, Sanofi, Eisai, Intra-Cellular, NewBridge Pharmaceuticals, Abbvie. RM is the CEO of Braxia Scientific Corp. KT has received personal fees from Braxia Scientific Corp. The remaining authors declare that the research was conducted in the absence of any commercial or financial relationships that could be construed as a potential conflict of interest.

## Publisher's Note

All claims expressed in this article are solely those of the authors and do not necessarily represent those of their affiliated organizations, or those of the publisher, the editors and the reviewers. Any product that may be evaluated in this article, or claim that may be made by its manufacturer, is not guaranteed or endorsed by the publisher.
