# Supplementary material for: Are Physical Activities Associated With Perceived Stress? The Evidence From the China Health and Nutrition Survey
Source: Front Public Health. 2021 Aug 3;9:697484. doi: 10.3389/fpubh.2021.697484 (PMC8369204; doi:10.3389/fpubh.2021.697484)
Supplement: Supplementary file 1 [file Table_1.DOCX]

**Supplemental Table 1 Metabolic equivalent of task (MET) intensity values of different physical activities**

| **Physical activity major headings** | **Specific activities** | **METS** |
| --- | --- | --- |
| **Occupational-related physical activity** | light activity at work | 1.8 |
|  | moderate activity at work | 4.5 |
|  | heavy activity at work | 6.5 |
| **Sports-related physical activity** | martial arts | 3.3 |
|  | gymnastics | 4.2 |
|  | track & field/swimming | 7.2 |
|  | walking | 4.0 |
|  | soccer/basketball/tennis | 5.5 |
|  | badminton/volleyball | 5.5 |
|  | others (Tai Chi, table tennis) | 3.3 |
| **Transport-related physical activity** | bus/ subway | 3.5 |
|  | bike | 6.8 |
|  | foot | 4.0 |
|  | car/taxi | 1.7 |
